# Early Phase of Specific Cellular Immune Status Associates with HCV Infection Outcomes in Marmosets

**DOI:** 10.3390/v15051082

**Published:** 2023-04-28

**Authors:** Bochao Liu, Enhui Zhang, Xiaorui Ma, Shengxue Luo, Chong Wang, Ling Zhang, Wenjing Wang, Yongshui Fu, Jean-Pierre Allain, Chengyao Li, Tingting Li

**Affiliations:** 1 Department of Transfusion Medicine, School of Laboratory Medicine and Biotechnology, Southern Medical University, Guangzhou 510515, China; 2 Guangzhou Blood Center, Guangzhou 510095, China; 3 Division of Transfusion Medicine, University of Cambridge, Cambridge CB2 2PT, UK

**Keywords:** chimeric HCV, infection outcome, early phase infection, immune mechanism, marmoset model

## Abstract

The major mechanism for determination of HCV infection outcomes has not been fully described, particularly in the early phase of the “window-period” of infection. Based on two groups of marmosets infected with HCV-CE1E2p7/GBV-B chimeric virus (HCV chimera) or GBV-B, the immune mechanism correlating with the different outcomes of virus infections was explored in this study. HCV chimera containing the entire HCV core and envelope proteins (CE1E2p7) and GBV-B RNA were intrahepatically injected into four marmosets in each group, respectively. Blood samples were taken from individual animals in an interval of 2 weeks. Viral load and specific T cell responses were detected in two groups of HCV chimera- and GBV-B-infected marmosets. HCV chimera-infected marmosets appeared to have a virally persistent infection over 6 months post inoculation of the virus. Of these, the specific IFN-γ-secretion T cell response slowly developed over 13 to 19 weeks and was maintained at a relatively low level with 40–70 SFC/10^6^ PBMCs, while the specific Treg cell response was rapidly activated over 3 weeks and was maintained at a high level around 5% among lymphocytes. In contrast, GBV-B-infected marmosets presented spontaneous viral clearance within 6 months; the specific IFN-γ-secretion T cell response was quickly established over 5 to 7 weeks and was maintained at a high level with 50–130 SFC/10^6^ PBMCs, while the specific Treg cell response was inactivated and maintained at a baseline below 3% among lymphocytes. In conclusion, the HCV structural proteins inducing immune suppression in the early phase of HCV infection contributed to the viral persistence, of which the activation of Treg cells might play an important role in the inhibition of an effective T cell antiviral response.

## 1. Introduction

The hepatitis C virus (HCV) causes hepatitis C, one of the viral liver diseases, which affects approximately 71 million people worldwide [1]. HCV infection usually has two types of acute and chronic outcomes, of which the acute infection normally spontaneously clears the virus, but the chronic infection becomes virally persistent [2]. Clinically chronic HCV infection may take decades to develop into advanced liver disease. Long-term chronic HCV infection can also cause glomerulonephritis, diabetes, thyroid disease, delayed skin porphyria, mixed cryoglobulinemia, lichen planus, and B-cell lymphoproliferative diseases [3]. Chronic HCV infection is often accompanied by a number of immune disorders, including liver inflammation, steatosis, and progressive fibrosis [4]. A small number of HCV infections develop into liver cirrhosis or hepatocellular carcinoma (HCC). Therefore, HCC may be caused by the progression of chronic inflammation, insulin resistance (IR), hepatocyte production, oxidative stress, fibrosis, and liver damage caused by chronic HCV infection [5].

In addition to interferon-based therapies, the direct-acting antiviral agents (DAAs) on HCV have been developed in recent years, which specifically inhibit the function of viral proteins necessary for viral replication [6]. These DAAs include NS3/4A protease inhibitors, NS5A replication complex inhibitors, nucleoside NS5B polymerase inhibitors, and non-nucleoside NS5B polymerase inhibitors [7]. Although these new drugs have a significant therapeutic effect of sustained antiviral response (SVR), they still cannot eradicate the virus completely, and drug-resistance and reinfection are increasingly reported [8]. HCV infection has a long-term asymptomatic characteristic, and in order to ultimately control the infection, an effective preventive vaccine needs to be developed. To achieve this goal, the mechanism for spontaneous viral clearance or persistence needs to be better understood to provide clues for protective immunity. However, as it is difficult or almost impossible to know the virus and host interaction during the very early phase of infection or the so-called “window-period” infection in humans, the two types of outcomes of HCV clearance or persistence have not been fully explored.

Common marmosets (*Callithrix jacchus*), one of the New World small primates, are susceptible to the GB virus B (GBV-B), a flavivirus phylogenetically related to HCV [9,10]. Marmosets exposed to GBV-B develop the self-limited infection similar to the spontaneously resolved HCV infection in humans [11,12]. In our previous study, we constructed an HCV-CE1E2p7/GBV-B chimeric virus (simplified as HCV chimera) containing the whole structural proteins (core-E1-E2-p7) of HCV, which could persistently infect the marmosets, resulting in typical viral hepatitis [13]. By using the marmosets infected with HCV chimera and GBV-B as animal models, this study investigates the immune mechanism leading to different outcomes of virus infection from initial exposure of viruses to animals.

## 2. Materials and Methods

### 2.1. Ethics Statement

Common marmoset experimentation and sample collection were approved by Southern Medical University (SMU) Animal Care and Use Committee (permit numbers: SYXK(Yue)2010-0056). All animal care and procedures (NFYYLASOP-037) were in accordance with national and institutional policies for animal health and well-being. All efforts were made to minimize suffering of animals.

### 2.2. Marmosets and Virus Inoculation

Eight common marmosets (male, age 2–3 years and weight 250–350 g) were imported from Tianjin Medical University and individually fed in the Laboratory Animal Research Center of Nanfang Hospital, Guangzhou, China. HCV-CE1E2p7/GBV-B chimeric RNA and GBV-B RNA were prepared by in vitro transcription with a MEGAscript kit according to manufactory instruction (Ambion, Applied Biosystems, Austin, TX, USA). Marmosets (*n* = 8) were randomly separated into four animals for each group. An intrahepatic injection of 300 μg HCV/GBV-B chimeric RNA or GBV-B RNA were injected into animals’ livers at two points, respectively, as in a previously described method [13]. Blood samples were collected from marmosets via femoral vein at an interval of 2 weeks. At the end point of the experiment, liver tissues were collected specifically for this study from HCV chimera- or GBV-B-infected animals.

### 2.3. RT-qPCR for Viremia Detection

Viral RNA was extracted from sera of infected marmosets using the High Pure Viral Nucleic Acid kit (Roche Diagnostic GmbH, Mannheim, Germany). Viremic or hepatocytic RNA of HCV chimera- or GBV-B-infected marmosets was measured by RT-qPCR with primers targeting the GBV-B 5′ NCR as previously described [13].

### 2.4. Biochemical Test

Serum alanine aminotransferase (ALT) and aspartate aminotransferase (AST) levels in the marmosets were measured in units per liter (U/L) by the commercial kits (Biosino Bio-technology and Science, Beijing, China) on a Hitachi 7170S fully automatic biochemical analyzer (Tokyo, Japan) [13].

### 2.5. Histopathological Examination

Small sections of liver tissue from each animal’s liver were examined with hematoxylin and eosin (H&E) staining as described previously [13]. The necrosis and inflammation were graded on the scales 0–18 according to the modified histology activity index (HAI) system [14].

### 2.6. ELISpot Assay

HCV and GBV-B peptides (Table S1) were selected by predicting peptide binding to Macaque MHC class I molecules from a website (http://www.mamu.liai.org/) accessed on 5 June 2014, which were commercially synthesized by the Chinese Peptide Company (Hangzhou, Zhejiang, China). HCV Chimera or GBV-B peptides (4 μg/mL each) were pooled in PBS. The specific IFN-γ-secretion T cell response of peripheral blood mononuclear cells (PBMCs) from marmosets was measured by stimulation with the pooled peptides in an enzyme-linked immunosorbent spot assay (ELISpot) as described previously [13]. Briefly, a pre-coated human gamma interferon ELISpot Plus plate (Mebtech AB, Nacka Strand, Sweden) was used in the assay. PBMCs were isolated from blood samples of marmosets and cultured in the pre-coated ELISpot plates at 3 × 10^5^ cells/well in 10% fetal calf serum, 100 IU/mL penicillin, and 100 IU/mL streptomycin 1640 medium containing pooled peptides and 100 U/mL interleukin-2 (IL-2). The negative and positive controls were 1640 medium or phytohemagglutinin (PHA), respectively. The result was normalized as the numbers of spot-forming cells (SFCs) per 10^6^ PBMCs.

### 2.7. Flow Cytometry

Freshly separated marmosets’ PBMCs were examined for regulatory T (Treg) cells by incubating for 30 min at room temperature in 0.1% BSA PBS containing anti-CD4-PE (IgG2a, Cat: MHCD0404, clone: s3.5, Invitrogen, Carlsbad, CA, USA) for 5 μL/sample and anti-Foxp3-APC (Mouse IgG2a kapa, Clone: PCH101, eBioscience, San Diego, CA, USA) for 2 μL/sample. Propidium iodide was added after the final wash at 1 mg/mL to exclude dead cells in all experiments. Samples were analyzed on a Becton Dickinson (San Jose, CA, USA) FACSort apparatus. Ten thousand events were collected and analyzed using CellQuest software version 3.3 (Becton Dickinson, San Jose, CA, USA).

### 2.8. Measurement of Cytokines

PBMCs (1 × 10^6^ per well) were stimulated for incubation for 5 days at 37 °C and 5% CO_2_ with peptides, 1640 medium, and PHA controls, respectively. Afterwards, the IFN-γ in the supernatants of cell cultures were measured by the human IFN-γ ELISA kit (U-Cytech Biosciences, Utrecht, Netherland).

Total RNA of PBMCs were extracted using TRIzol method (Invitrogen) and reverse-transcripted using Reverse Transcription System according to the manufacturer’s instructions (Roche, Basel, Switzerland). Quantitative RT-PCR reactions for IL-10 and TGF-β mRNA transcripts were performed with SYBR Master Mix following the manufacturer’s protocol (Roche, Basel, Switzerland). Specific IL-10 primers were 5′-CTGCCTCACATGCTTCGAGA-3′ (forward) and 5′-TGGCAACCCAGGTAACCCTTA-3′ (reverse), and TGF-β primers were 5′- GGGTTCTCTTGGCTGTTACTG-3′ (forward) and 5′-TGTCTAAGAAAAGAGTTCCATTATC-3′ (reverse), respectively.

### 2.9. Statistical Analysis

All experiments were repeated at least three times independently. The data were analyzed using the statistical package SPSS v. 19.0. The results were presented as the mean ± SD. Differences between groups were analyzed using Student’s *t*-test, and *p*-values < 0.05 were considered statistically significant.

## 3. Results

### 3.1. Different Outcome of Viral Persistence from HCV Chimera or Clearance from GBV-B-Infected Marmosets

**Figure 1:**
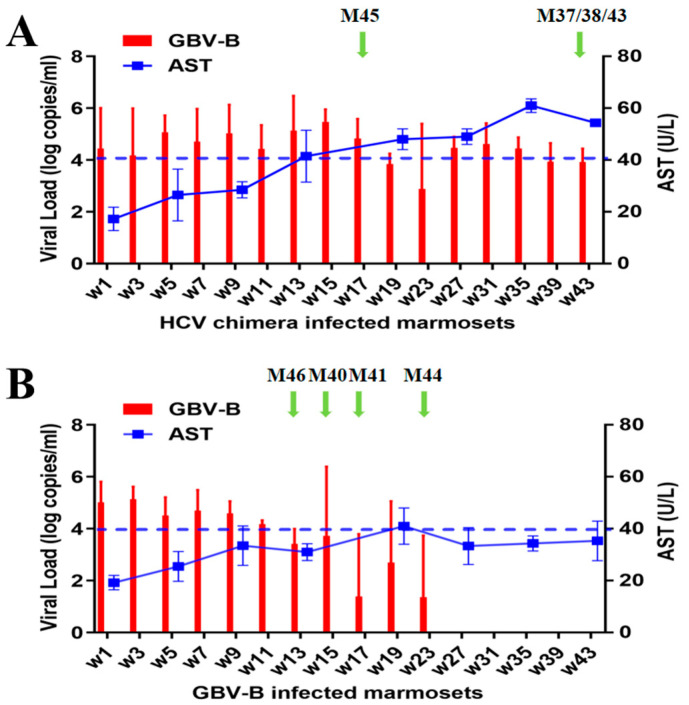
Detection of viremia in HCV chimera- or GBV-B-infected marmosets by RT-qPCR. Viral load (VL, log10 copies/mL) and AST (U/L) in serum sample of HCV chimera-infected marmosets (**A**) and GBV-B-infected marmosets (**B**) was tested at an interval of 2 weeks from two groups of animals (overall). The endpoints of monitoring animals are indicated by green arrows.

Four marmosets, M37, M38, M43, and M45, were infected with HCV-CE1E2p7/GBV-B chimeric virus (HCV chimera), and four marmosets, M40, M41, M44, and M46, were infected with GBV-B, respectively. After intrahepatic injection of viral genomic RNA, 2 mL of blood samples were collected at an interval of two weeks from all marmosets. Viral replication was measured by detecting viremic RNA with RT-qPCR (Figure 1). Among the HCV chimera-infected group, marmosets M37, M38, and M43 were examined for 43 weeks, while M45 died 17 weeks after infection (Figure S1A). An overall pattern of viral replication exhibited a typical persistent infection with viral loads ranging between 7.41 (±2.37) × 10^2^ and 2.95 (±0.48) × 10^5^ copies/mL (Figure 1A). In the GBV-B-infected group, marmosets M41, M44, and M46 were examined for 43 weeks, and M40 died 15 weeks after infection (Figure S1B), of which an overall pattern showed a self-limited infection with viral loads ranging between 24 (±2.41) and 1.34 (±0.49) × 10^5^ copies/mL and then the virus spontaneously cleared in 23 weeks (Figure 1B). The results suggested that HCV chimera-infected marmosets progressed to chronic infection, while GBV-B-infected marmosets resolved their infections naturally.

The serum ALT was detected within a normal range (<40 U/L) from both HCV chimera- and GBV-B-infected marmosets. The AST was progressively elevated over 40 U/L in the serum of HCV chimera-infected marmosets during the course of detection, while the AST was mostly stable below 40 U/L in the serum of GBV-B-infected marmosets (Figure 1 and Figure S1).

### 3.2. HCV Chimera Was More Pathogenic to Marmosets Than GBV-B

**Figure 2:**
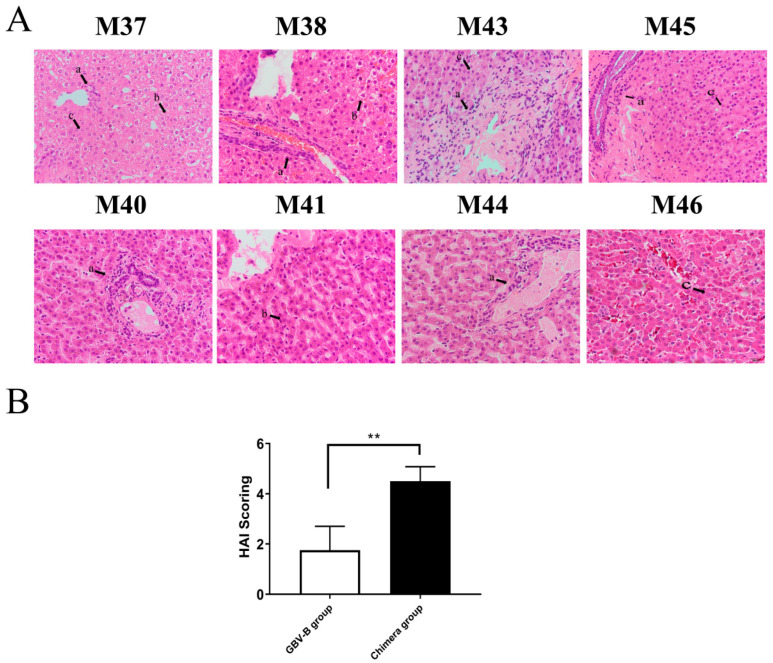
Hematoxylin and eosin staining of liver tissues from HCV CE1E2p7 chimera- or GBV-B-infected marmosets. (**A**) HCV chimera-infected marmosets M37, M38, M43, and M45; GBV-B-infected marmosets M40, M41, M44, and M46. (**B**) HAI scoring comparison between two groups of animals. (**a**) Lymphocytic infiltrates, (**b**) ballooning degeneration (edema), (**c**) ground glass liver cells. Necroinflammatory grades in histopathological changes in liver tissues were scored by the modified HAI system, in which inflammation grades were on a scale of 0 to 18. ** *p* < 0.01.

The histopathologic examination for liver tissues was conducted from HCV chimera- and GBV-B-infected marmosets at the end point of follow-up detection (Figure 2; Table S2). The viral load was measured for 10^3^–10^4^ copies/mg in liver tissue from HCV chimera-infected marmosets, but not from GBV-B-infected animals except for animal M40 that died at week 15 (Figure S2). The HCV chimera-infected marmosets presented typical viral hepatitis in the liver tissues, such as lymphocytic infiltration, ballooning, ground-glass changes, and eosinophilic changes, while the GBV-B-infected animals exhibited mild viral hepatitis in liver tissues (Figure 2A). The results suggested that HCV-CE1E2p7 chimera persistent infection caused more severe pathological changes than GBV-B resolved infection in marmosets (Figure 2B).

### 3.3. GBV-B Infection Induced Significantly Higher IFN-γ-Secretion T Cell Response Than HCV Chimera Infection in Marmosets

**Figure 3:**
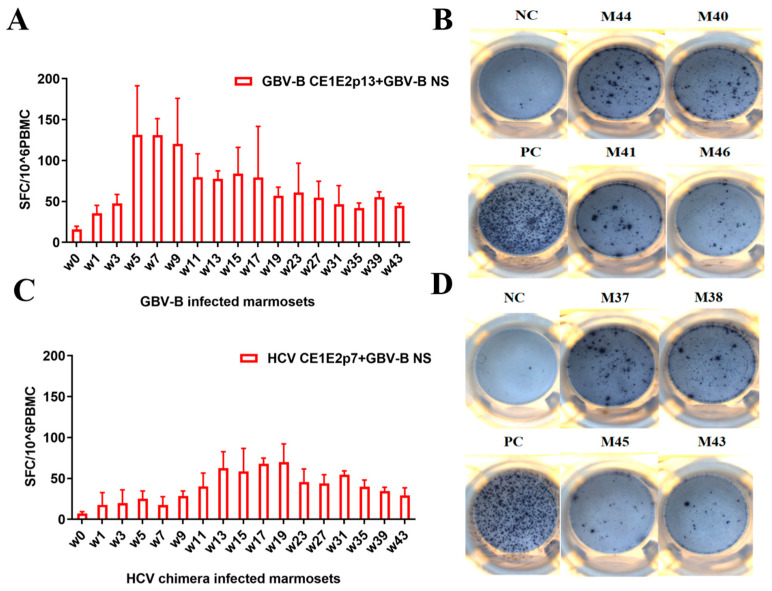
The specific T cell response in two groups of marmosets infected with HCV chimera or GBV-B. (**A**,**B**) IFN-γ-secretion T cell response from GBV-B-infected marmosets detected by ELISpot. The stimulating peptides were derived from GBV-B structural and non-structural proteins. (**C**,**D**) IFN-γ-secretion T cell response from HCV chimera-infected marmosets detected by ELISpot. The stimulating peptides were derived from HCV structural and GBV-B non-structural protein. The detection time point of representative results in the figure panels B and D was at week 43 for marmosets M37, M38, M43, M41, M44, and M46, or at week 15 for M45 and M40, respectively.

In order to characterize the specific cellular immune responses between HCV chimera- and GBV-B-infected marmosets, the PBMCs were freshly isolated from these two different virus infected animals, and the specific IFN-γ-secretion T cell responses were detected by ELISpot (Figure 3 and Figure S3). In four GBV-B-infected marmosets, the specific IFN-γ-secretion T cell response increased rapidly to the high level (>100 SFC/10^6^ PBMCs) at weeks 5–7 after infection and was then maintained at a relatively high level > 50 SFC/10^6^ PBMCs (Figure 3A,B). In HCV chimera-infected marmosets, the IFN-γ-secretion T cell response slowly increased to the plateau (<100 SFCs/10^6^ PBMCs) at weeks 13–19 after infection and was then maintained at a low level < 50 SFCs/10^6^ PBMCs (Figure 3C,D). The results suggested that HCV chimera-infected marmosets exhibited a delayed and lower specific T cell response, whereas GBV-B-infected marmosets showed a rapid and strong specific T cell response after the different virus infections.

### 3.4. HCV Chimera Infection Induced Significantly Higher Treg Cell Response Than GBV-B Infection in Marmosets

**Figure 4:**
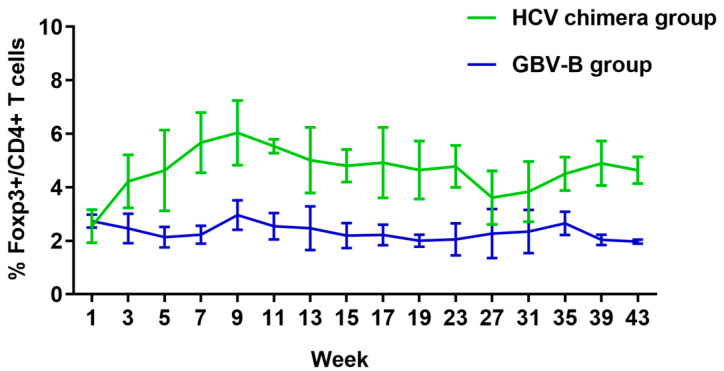
The percentage of Treg cells among lymphocytes in two groups of marmosets infected with HCV chimera or GBV-B. The percentage (%) of Treg cells in HCV chimera-infected group (green) and GBV-B-infected group (blue) of animals.

Further, to investigate the differences in suppressive immune response between the two different virus infected marmosets, the Treg cell response was measured by flow cytometry with Foxp3+/CD4+ biomarker antibodies (Figure 4 and Figure S4). In HCV chimera-infected marmosets, the ratio of Treg cells was rapidly increased to approximately 4% in 3 weeks, 6% at the top in 9 weeks, and then maintained for 4–6% at a high level until 43 weeks among lymphocytes after virus infection. In GBV-B-infected marmosets, Treg cell response was not activated and the ratio of Treg cells was around 2% at a baseline level before or after virus infection. The results suggested that HCV chimera infection rapidly activated and amplified the suppressive T cell response, while GBV-B infection did not induce a Treg response in the marmosets.

### 3.5. Activation of Treg Cell Response by HCV Structural Proteins

**Figure 5:**
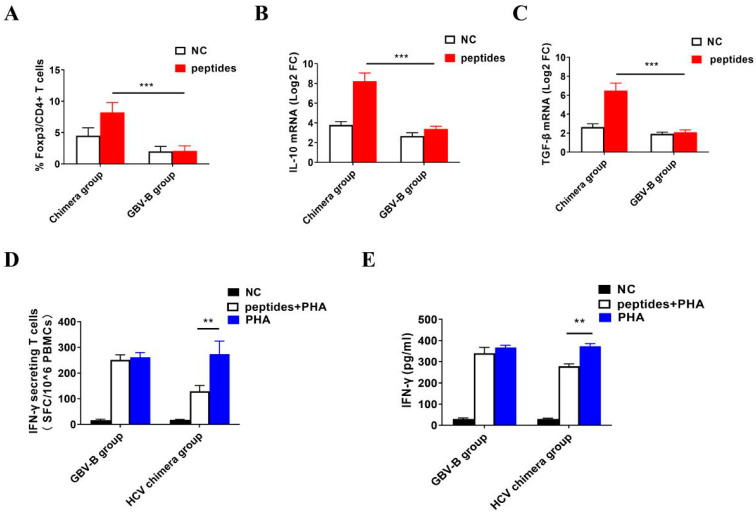
In vitro T cell responses from PBMCs stimulated with peptides or PHA. (**A**) The comparison of Treg cell frequencies in PBMCs stimulated with peptides in vitro between HCV chimera- and GBV-B-infected marmosets. The comparison of mRNA levels of IL-10 (**B**) or TGF-β (**C**) in PBMCs after peptide stimulation in vitro between HCV chimera- and GBV-B-infected marmosets. The level of mRNA was presented as log2 fold changes (FC). The comparison of IFN-γ-secreting T cells among PBMCs (**D**) or secretion IFN-γ in the supernatant of PBMCs (**E**) by stimulation with HCV or GBV-B whole structural protein peptides plus PHA and PHA only from HCV chimera- or GBV-B-infected marmosets. IFN-γ-secretion T cells (SFC/10^6^ PBMCs) and secretion IFN-γ (pg/mL) were measured by ELISpot or ELISA, respectively. ** *p* < 0.01, *** *p* < 0.001.

To verify the activation of Treg cells by HCV chimera via its structural proteins, the specific Treg cell response was measured in vitro by stimulating the marmosets’ PBMCs with HCV structural protein peptides in comparison with GBV-B peptides, respectively (Figure 5 and Figure S5). From HCV chimera-infected marmosets (Figure S5A), the mean ratio of Treg cells was detected at 8.5% among PBMCs stimulated with HCV structural protein peptides, which were significantly higher than that at 4.6% in negative controls (Figure 5A; *p* < 0.001). From GBV-B-infected marmosets (Figure S5B), the mean ratio of Treg cells was detected at 2% among PBMCs stimulated with GBV-B structural protein peptides, which was not significantly different from negative controls (Figure 5A; *p* > 0.05). The ratio of Treg cells in HCV peptide-stimulated PBMCs was higher than that in GBV-B peptide-stimulated PBMCs from two different virus infected marmosets (Figure 5A; *p* < 0.001). The results suggested that HCV whole structural proteins could specifically activate Treg cells in PBMCs from HCV chimera-infected marmosets, whereas GBV-B whole structural proteins could not activate Treg cells in PBMCs from GBV-B-infected marmosets. At the mRNA level, IL-10 and TGF-β transcripts were higher in HCV peptide-stimulated PBMCs than those in GBV-B peptide-induced cells (Figure 5B,C; *p* < 0.001).

### 3.6. Inhibition of IFN-γ-Secretion T Cell Response by Activated Treg Cells

To investigate whether the low level of IFN-γ-secretion T cell response in HCV chimera-infected marmosets was inhibited by the early activated Treg cells, the PBMCs were stimulated correspondingly by the negative control (NC), HCV structural protein peptides plus PHA, and PHA only, respectively. The IFN-γ-secretion T cell response was measured as 17 ± 4 SFC (NC), 129 ± 15 SFC (HCV peptides + PHA), and 273 ± 47 SFC (PHA only) per million PBMCs, respectively (Figure S6A). Comparably, by stimulating PBMCs from GBV-B-infected marmosets with NC, GBV-B structural protein peptides plus PHA, and PHA only, IFN-γ-secretion T cell responses were measured as 16 ± 3 SFC, 250 ± 27 SFC, or 261 ± 33 SFC/10^6^ PBMCs, respectively (Figure S6B). The data showed that IFN-γ-secretion T cell response induced by HCV peptides plus PHA was lower than that induced by PHA only (Figure 5D; *p* < 0.01), while IFN-γ-secretion T cell response had no significant differences between GBV-B peptides plus PHA and PHA only inductions (Figure 5D; *p* > 0.05), suggesting that the HCV structural protein might suppress IFN-γ-secretion T cell response. By quantifying the secreted IFN-γ in supernatants of stimulated PBMCs from HCV chimera- or GBV-B-infected marmosets, IFN-γ was significantly decreased in simulation with HCV peptides plus PHA than that with PHA only (Figure 5E; *p* < 0.01). Overall, the data suggested that HCV structural proteins inhibited specific IFN-γ-secreting T cell responses via activation of Treg cells, but GBV-B did not activate Treg cells in the virus-infected marmosets.

## 4. Discussion

Hepatitis C is a blood transmitted and serious infectious disease caused by HCV, which has a prevalence rate of 2.8% globally [15]. HCV infection usually progresses towards two outcomes: acute infection leading to spontaneous virus clearance and chronic infection developing to viral persistence. Among HCV infections, 55–85% develop to chronicity, with 10% of them progressing to cirrhosis and HCC eventually [16,17]. What factors lead to different outcomes from HCV infection? We tried to explore the mechanism of immune responses at the early phase of HCV chimera- or GBV-B-infected marmosets for differentiating viral persistence and resolution in this study.

GBV-B is a hepatovirus of flaviviridae discovered in 1995 with 28% amino acid similarity to genotype 1 of HCV. Marmosets are susceptible to GBV-B but have acute self-limited infection with viral clearance within 20 weeks after exposure to the virus [18]. However, HCV chimera carrying whole structural proteins (core, E1, E2, and p7) of HCV is able to infect marmosets persistently [13]. Therefore, HCV chimera- and GBV-B-infected marmosets provided an ideal surrogate animal model to realize the immune mechanism of HCV infection progression.

A large number of studies have focused on the mechanisms on outcomes of resolution or persistence from HCV infection in humans [19,20,21,22,23]. However, characterization in the early phase of HCV infection is still extremely scarce due to the unknown exposure time to humans. With the advantage of HCV chimera- or GBV-B-infected marmoset models (Figure 1), therefore, we could monitor the immune responses of the early phases from two groups of marmosets after intrahepatic injection of the virus. Most importantly, a significant difference in specific T cell responses was found between HCV chimera- and GBV-B-infected animals (Figure 3). GBV-B-infected marmosets quickly produced a strong specific T cell response in 5 weeks, while HCV chimera-infected marmosets slowly developed a low level of T cell response (only half of the GBV-B group) in about 13 weeks after virus exposure to the animals. A previous study has shown that the clearance of GBV-B in marmosets is closely related to cellular immunity, especially specific T cell responses [18]. In our study, we additionally found that the frequency of Treg cells varied significantly between HCV chimera- and GBV-B-infected marmosets (5% vs. 2%; Figure 4).

CD4+Foxp3+ regulatory T (Treg) cells constitute a subset of specific suppressor T cells, which can inhibit the activation and proliferation of various types of immune cells including T cells, B cells, NK cells, and dendritic cells [24]. Treg cells play an important role in maintaining immune tolerance by inhibiting autoreactive T cells, and control excessive immune activation after the infection of various pathogens [25]. In addition, Treg cells also play an important role in regulating antiviral-specific T cell responses and immune-mediated host injury in the acute and chronic stages of viral infection [26]. Treg cells can modulate effector T cell responses against pathogens such as the herpes simplex virus (HSV), human immunodeficiency virus (HIV), and simian immunodeficiency virus (SIV) [27]. An effective virus-specific T cell response is essential for elimination of the virus, and during this process Treg cells inversely inhibit the antiviral-specific T cell response, thus facilitating persistent infection of the virus. The kinetic role of Treg cells has rarely been studied in acute HCV infections due to the difficulty in capturing the initial infection for follow-up detection. Previous studies have analyzed the differences in circulating Treg cells between patients with acute HCV infections who were ultimately converted to chronic infections or spontaneously cleared infections [28]. The results indicated that chronic infection was associated with long-term maintenance of Treg cells, whereas viral self-clearing in the acute phase was associated with a temporary loss of Treg cell suppressor function.

To further investigate the causes of the difference in specific T cell responses between HCV chimera- and GBV-B-infected marmosets, we measured the frequency of Treg cells among the lymphocytes from two groups of eight animals (Figure 4). The results showed that the frequency of Treg cells from four HCV chimera-infected marmosets rapidly rose to the high level in 3 weeks, to the top in 9 weeks, and then remained at a high level until 43 weeks during the course of follow-up detection. In contrast, Treg cells from four GBV-B-infected marmosets were insignificantly varied at the baseline. By stimulating PBMCs with HCV or GBV-B peptides, the proliferation response of Treg cells was observed as being significantly higher from HCV chimera-infected marmosets than that from GBV-B-infected marmosets (Figure 5A). In addition, the higher IL-10 and TGF-β mRNA transcripts were also detected from HCV chimera but not from GBV-B-induced marmosets’ PBMCs (Figure 5B,C). The data suggested that Treg cell response was activated, proliferated, and maintained since the early phase of HCV chimera infection in marmosets, but inactivated and stabilized at the baseline during the course of GBV-B infection in the marmosets, which might be associated with the levels of effective T cell response for the progression of HCV infection.

To understand the different outcomes from these two different virus infections, a hypothesis was focused on the difference in structural proteins of HCV chimera and GBV-B. Based on the GBV-B genomic backbone, the structural protein genes (CE1E2p13) of GBV-B are replaced by the corresponding region of HCV CE1E2p7 genes, while the non-structural protein regions of the two viruses are identical. However, GBV-B-infected marmosets showed a prompt and highly effective T cell response and an inactivated and low suppressive T cell response, while, conversely, the HCV chimera-infected marmosets presented a delayed and lower effective T cell response and an activated and highly suppressive T cell response (Figure 4). So, we hypothesized that the HCV structural proteins play an important role in immune suppression leading to the persistent infection of HCV. To test this hypothesis, we stimulated the specific IFN-γ-secreting T cell response of PBMCs in vitro with HCV structural protein peptides plus PHA or PHA only in comparison with GBV-B structural protein peptides plus PHA or PHA only, respectively. The results showed that HCV structural protein peptides inhibited T cell response of PBMCs induced by PHA, but GBV-B structural protein peptides did not (Figure 5D,E), suggesting HCV structural proteins might play an important role in suppression of effective T cell response through the activation of the Treg cell response. A study on chronically infected hepatitis C patients has shown that the HCV core protein triggers Treg activation and expansion to inhibit CD4+ T cell proliferation and IFN-γ secretion in a Treg-dependent manner, which may play a critical role in viral persistence in HCV-infected patients [29]. To further demonstrate whether or not the IFN-γ-secretion T cell response is suppressed by HCV core epitopes via Treg cells (Figure 5), the depletion of Treg cells is suggested for performing the experiment in the future. However, the epitopes within the HCV core protein inducing the Treg cell response need to be further identified. In line with immune suppression in HCV CE1E2p7 chimera-infected marmosets, the more severe pathological changes in hepatitis were observed by comparison with GBV-B infection in this study or HCV E1E2p7 chimera infection in our previous study [13], which also explored that the HCV core protein induced an increase in IL-32 expression via the PI3K pathway in hepatic cells leading to HCV-related severe hepatitis [30].

## 5. Conclusions

In conclusion, the specific cellular immune status in the early phase of HCV infection critically contributes to the different outcomes, of which the HCV core protein may activate Treg cells to inhibit an effective T cell response leading to viral persistence, or otherwise viral clearance.

## Data Availability

All data are available from the main article and supplemental information.

## Supplementary Materials

The following supporting information can be downloaded at: https://www.mdpi.com/article/10.3390/v15051082/s1, Figure S1: Detection of viremia or AST in serum from HCV chimera (A) and GBV-B infected marmosets (B); Figure S2: The quantification of viral load in liver tissues from the endpoint of HCV chimera or GBV-B infected marmosets; Figure S3: The specific IFN-γ secreting T-cell response of PBMCs (SFC/10^6^ cells) detected from HCV chimera (A) and GBV-B infected marmosets (B); Figure S4: The percentage of Treg cells among lymphocytes from HCV chimera (A) and GBV-B infected marmosets (B); Figure S5: Frequency of Treg cells in PBMCS from HCV chimera or GBV-B infected marmosets was measured by flow cytometry; Figure S6: IFN-γ secretion T cell response was measured in PBMCs stimulated with HCV or GBV-B structural protein peptides plus PHA or PHA only; Table S1: HCV and GBV-B peptides used in ELISpot assay; Table S2: Histopathological scoring of liver tissues from HCV chimera and GBV-B infected marmosets.
