# Supplementary material for: Early Phase of Specific Cellular Immune Status Associates with HCV Infection Outcomes in Marmosets
Source: Viruses. 2023 Apr 28;15(5):1082. doi: 10.3390/v15051082 (PMC10222972; doi:10.3390/v15051082)
Supplement: Supplementary file 1 [file viruses-15-01082-s001.zip › viruses-2337002-supplementary.pdf]

# Early phase of specific cellular immune status associates with HCV infection outcomes in marmosets

Bochao Liu[1,2*], Enhui Zhang[1*], Xiaorui Ma[1], Shengxue Luo[1], Chong Wang[1], Ling Zhang[1], Wenjing Wang[1], Yongshui Fu[2], Jean-Pierre Allain[3], Chengyao Li[#1], Tingting Li[#1]

[1] Department of Transfusion Medicine, School of Laboratory Medicine and Biotechnology, Southern Medical University, Guangzhou, China;

[2] Guangzhou blood center, Guangzhou, China;

[3] Emeritus professor of transfusion medicine, University of Cambridge, UK.

* These authors contributed equally to this work.

[#] **Corresponding author:** Tingting Li or Chengyao Li, Department of Transfusion Medicine, School of Laboratory Medicine and Biotechnology, Southern Medical University, No. 1838 North Guangzhou Avenue, Guangzhou, China.

Email: apple-ting-007@163.com (T Li); or chengyaoli@hotmail.com (C Li). Phone: 0086-20-61649360

**Table S1**. HCV and GBV-B peptides used in ELISpot assay

| No. | Sequence | Location |
|---|---|---|
| HCV | | |
| Core | | |
| 1 | RGPRLGVRA | 40-48 |
| 2 | AQPGYPWPL | 77-85 |
| 3 | GYPWPLYGN | 80-88 |
| 4 | GSRPSWGPS | 102-110 |
| 5 | APLGGVARA | 142-150 |
| 6 | ARALAHGVR | 148-156 |
| 7 | GVRVLEDSV | 154-162 |
| E1 | | |
| 8 | DSVNYATGN | 160-168 |
| 9 | NLPGCSFSI | 168-176 |
| 10 | LTIPASAYE | 185-193 |
| 11 | TIPASAYEV | 186-194 |
| 12 | LTPTLAARN | 242-250 |
| 13 | ARNSSVPTK | 248-256 |
| 14 | PTKTIRRHV | 254-262 |
| 15 | SQLFTFSPR | 288-296 |
| 16 | FSPRRHETV | 293-301 |
| 17 | ETVQDCNCS | 299-307 |
| 18 | NCSLYPGHV | 305-313 |
| 19 | GHVSGHRMA | 311-319 |
| 20 | RMAWDMMMN | 317-325 |
| 21 | MNWSPTAAL | 324-332 |
| 22 | RIPQAVVDM | 339-347 |
| E2p7 | | |
| 23 | FSLGPTQRI | 403-411 |
| 24 | GPTQRIQLV | 406-414 |
| 25 | ASCRPIDKF | 457-465 |
| 26 | SSDQRPYCW | 479-487 |
| 27 | TTDRSGVPT | 518-526 |
| 28 | TRPPQGNWF | 542-550 |
| 29 | RPPQGNWFG | 543-551 |
| 30 | GPPCNIGGV | 566-574 |
| 31 | SGPWLTPRC | 599-607 |
| 32 | MVDYPYRLW | 608-616 |
| 33 | TTLPALSTG | 680-688 |
| 34 | HLHQNIVDV | 691-699 |
| GBV-B | | |
| CoreE1E2p13 | | |

| | | |
|---|---|---|
| 1 | ISTQTSPVP | 4-12 |
| 2 | TSPVPAPRT | 8-16 |
| 3 | QTQASYPVS | 21-29 |
| 4 | KSRNLGILL | 83-91 |
| 5 | LLDYPLGWI | 90-98 |
| 6 | DVTTHTPLV | 100-108 |
| 7 | HTPLVGPLV | 104-112 |
| 8 | VGPLVAGAV | 108-116 |
| 9 | ATGWFGVHL | 134-142 |
| 10 | TGWFGVHLF | 135-143 |
| 11 | CSPSTCLHE | 181-189 |
| 12 | YSPKWTRPI | 387-395 |
| 13 | VTPWLTTAW | 457-465 |
| 14 | DTPIVYFYD | 501-509 |
| 15 | RLPGTPPVV | 523-531 |
| 16 | VGPWOLVAL | 712-720 |
| 17 | GPWPLVALL | 713-721 |
| NS2-5B | | |
| 18 | KSDDPYWCV | 850-858 |
| 19 | HATDATTVL | 1023-1031 |
| 20 | VIPTPHANI | 1058-1066 |
| 21 | PTPHANITE | 1060-1068 |
| 22 | LTDEGTIPF | 1071-1079 |
| 23 | CTPSGMVPE | 1209-1217 |
| 24 | RTQPGLPAI | 1246-1254 |
| 25 | RTADNYVLL | 1280-1288 |
| 26 | STITTTSPF | 1633-1641 |
| 27 | ITTPLPHKI | 1690-1698 |
| 28 | LTDARGALA | 1713-1721 |
| 29 | HTPGVRMQL | 2060-2068 |
| 30 | TTKLPAPSI | 2117-2125 |
| 31 | TATTASSYV | 2232-2240 |
| 32 | IVPKEEVFV | 2412-2420 |
| 33 | ITPEDIMVE | 2499-2507 |
| 34 | LSDQHRAGI | 2516-2522 |
| 35 | CVPQPKYSL | 2623-2631 |
| 36 | KSGKPYYFL | 2647-2655 |
| 37 | LTRDPRIPL | 2655-2663 |
| 38 | YSPEGDVFI | 2825-2833 |

Note: these peptides were derived from the amino acid sequences of HCV-CE1E2p7 chimera (GenBank: KF285483.1) and GBV-B (GenBank: NC_001655.1) structural and non-structural proteins.

**Table S2.** Histopathological scoring of liver tissues from HCV chimera and GBV-B infected marmosets

| Virus | Marmoset | Time point (week) | Necroinflammatory Grade | | | |
|---|---|---|---|---|---|---|
| | | | Periportal +/- bridging necrosis | Intralobular degeneration and focal necrosis | Portal inflammation | Total |
| HCV chimera infected marmosets | M37 | W43 | 0 | 3 | 1 | 4 |
| | M38 | W43 | 1 | 3 | 1 | 5 |
| | M43 | W43 | 0 | 3 | 1 | 4 |
| | M45 | W17 | 0 | 4 | 1 | 5 |
| GBV-B infected marmosets | M40 | W15 | 0 | 1 | 1 | 2 |
| | M41 | W43 | 0 | 1 | 0 | 1 |
| | M44 | W43 | 0 | 2 | 1 | 3 |
| | M46 | W43 | 0 | 1 | 0 | 1 |

Note: Necroinflammatory grade was scored by the modified HAI system. The histological status was determined by the modified HAI system (Kondell score), which grades necrosis and inflammation on a scale of 0 to 18 (periportal inflammation and necrosis, 0 to 10; lobular inflammation and necrosis, 0 to 4; portal inflammation, 0 to 4).

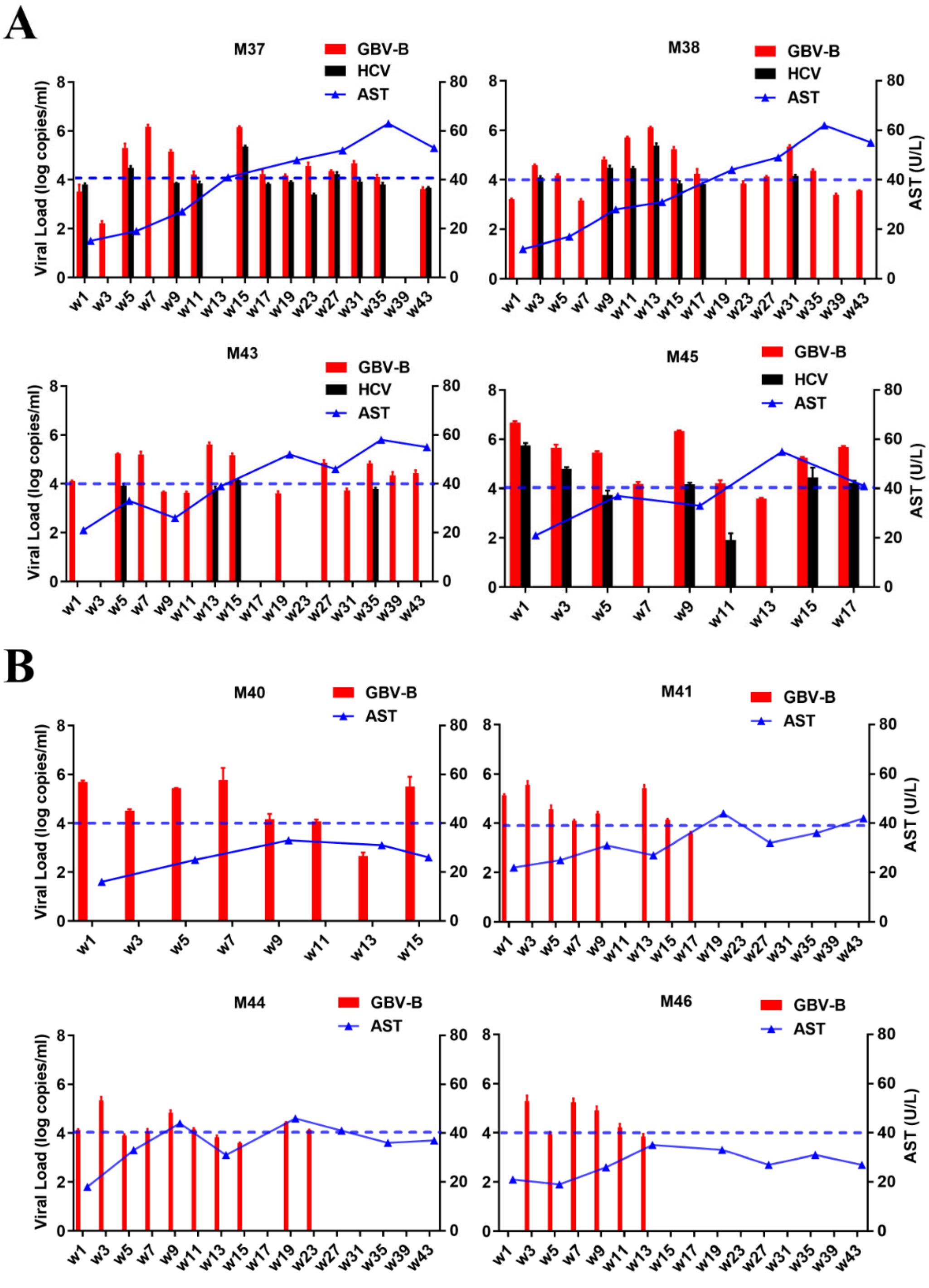


**Figure S1.** Detection of viremia or AST in serum from HCV chimera (A) and GBV-B infected marmosets (B). Viral RNA was measured by RT-qPCR with primers targeting the GBV-B 5' NCR (red) or HCV core (black).

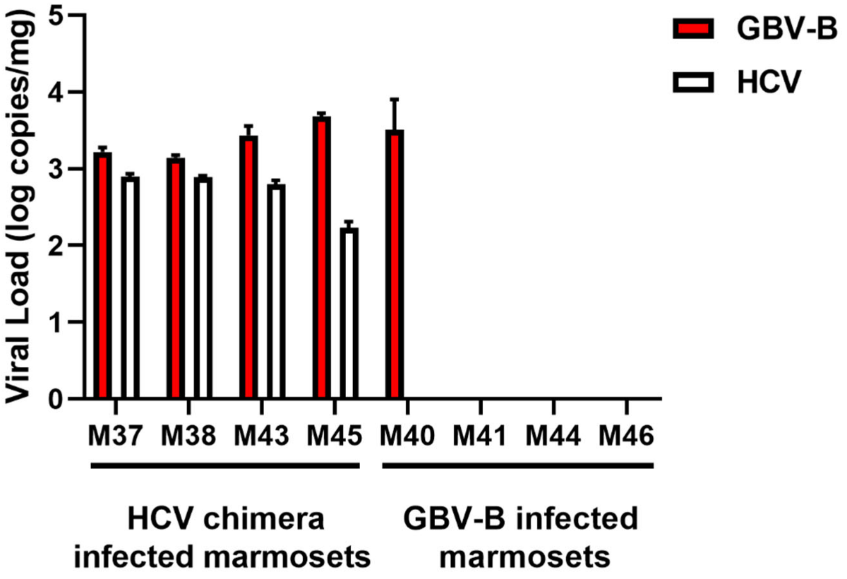


**Figure S2.** The quantification of viral load in liver tissues from the endpoint of HCV chimera or GBV-B infected marmosets. Hepatocyte viral load (log10 copies/mg) was quantified by RT-qPCR with primers targeting the GBV-B 5’ NCR (red) or HCV core (white).

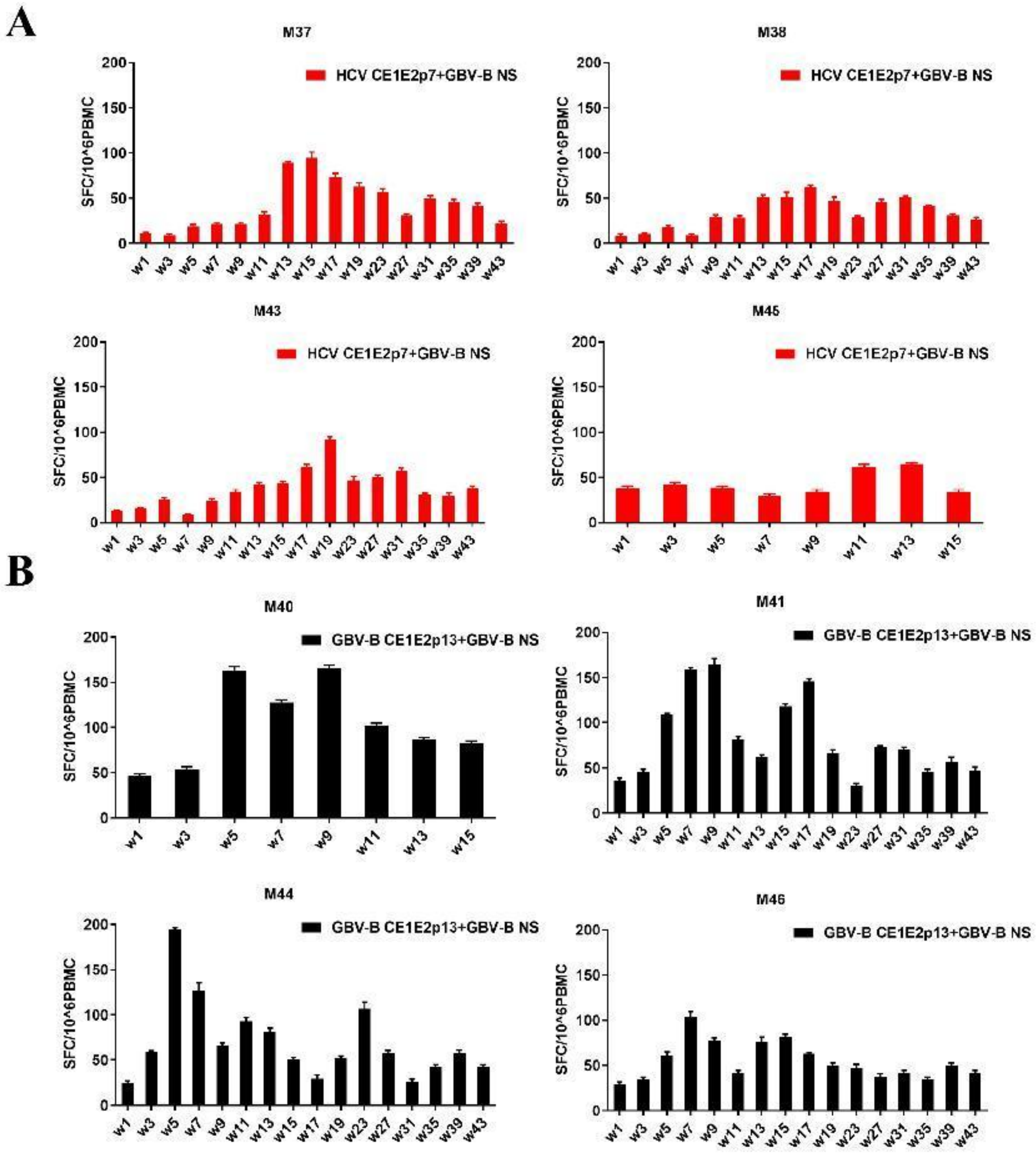


**Figure S3.** The specific IFN-γ secreting T-cell response of PBMCs (SFC/$10^6$ cells) detected from HCV chimera (A) and GBV-B infected marmosets (B).

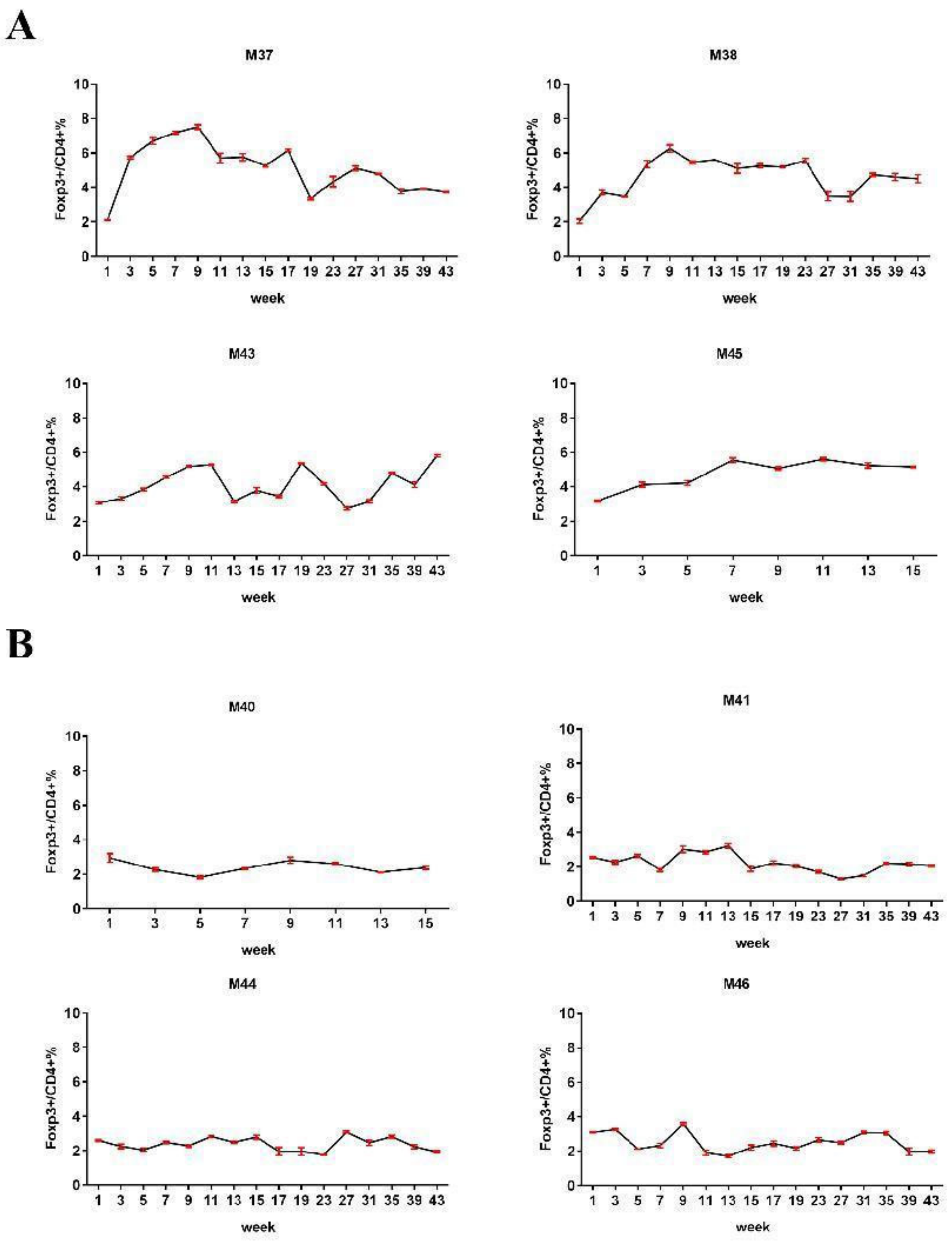


**Figure S4.** The percentage of Treg cells among lymphocytes from HCV chimera (A) and GBV-B infected marmosets (B).

.

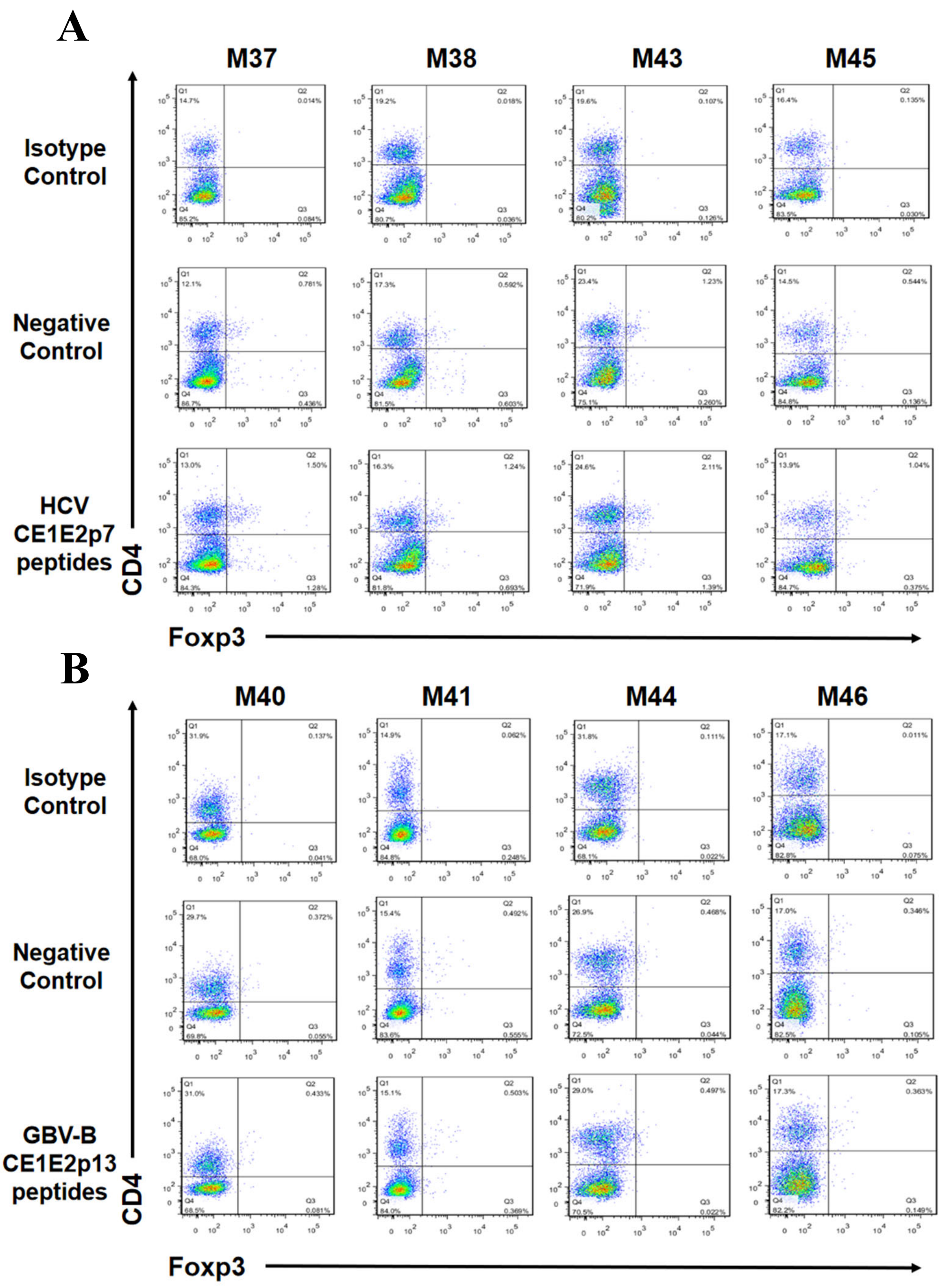


**Figure S5.** Frequency of Treg cells in PBMCS from HCV chimera or GBV-B infected marmosets was measured by flow cytometry. (A) Treg cells in PBMCs stimulated *in vitro* with HCV structural protein peptide pool from HCV chimera infected marmosets. (B) Treg cells in PBMCs stimulated *in vitro* with GBV-B structural protein peptide pool from GBV-B infected marmosets.

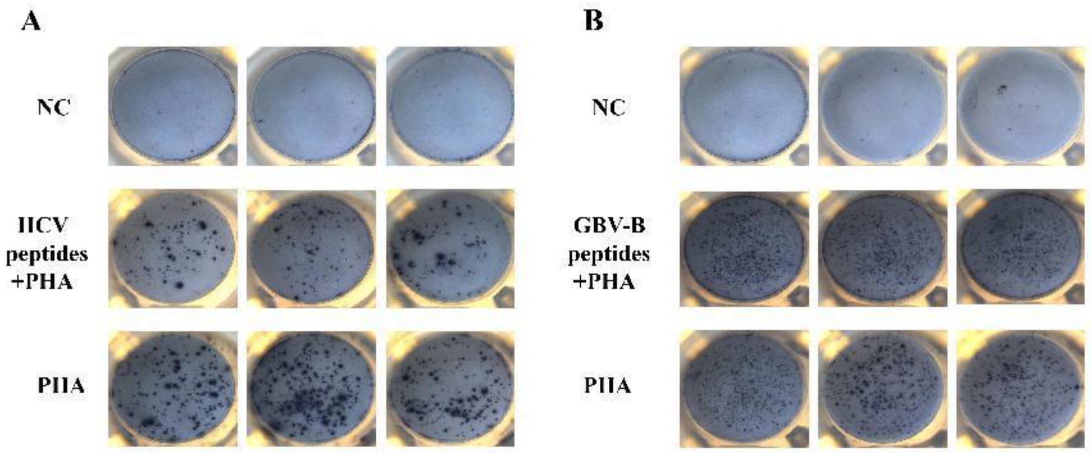


**Figure S6.** IFN-γ secretion T cell response was measured in PBMCs stimulated with HCV or GBV-B structural protein peptides plus PHA or PHA only. (A) Testing of IFN-γ secretion T cell response of PBMCs containing Treg cells from HCV chimera infected marmosets. (B) Testing of IFN-γ secretion T cell response of PBMCs containing Treg cells from GBV-B infected marmosets.
